# Supplementation of Acqua Lete® (Bicarbonate Calcic Mineral Water) improves hydration status in athletes after short term anaerobic exercise

**DOI:** 10.1186/1550-2783-9-35

**Published:** 2012-07-26

**Authors:** Paola Brancaccio, Francesco Mario Limongelli, Iride Paolillo, Antonio D’Aponte, Vincenzo Donnarumma, Luca Rastrelli

**Affiliations:** 1 Servizio di Medicina dello Sport, University of Naples, Via Costantinopoli, Naples, 16 80138, Italy; 2 Dipartimento di Scienze Farmaceutiche e Biomediche, University of Salerno, Via Ponte Don Melillo, Fisciano, Salerno, 84084, Italy; 3 Roecker Diagnostics and clinical research Laboratory, Marano di Napoli, Italy; 4 Dipartimento di Scienze Farmaceutiche e Biomediche, University of Salerno, Via Ponte Don Melillo, Fisciano, Salerno, 84084, Italy

**Keywords:** Acqua Lete® mineral water, Urine specific gravity, Urine pH, Intracellular body water, Muscular ultrasound

## Abstract

**Background:**

Experimental studies suggest that mineral waters with high concentrations of calcium and bicarbonate can impact acid–base balance. The purpose of this study was to test the effect on acid–base balance and specific urine gravity, of a bicarbonate calcic mineral water (Acqua Lete®) compared to a minimally mineralized water.

**Methods:**

88 amateur male athletes underwent two experimental trials with a modified Wingate test: the first was carried out without hydration (Control Test, Test C, n = 88); the second was carried out after one week of controlled hydration (Test with hydration, Test H, n = 88), with 1.5 L/day of a very low mineral content water (Group A, n = 44) or 1.5 L/day of Acqua Lete® (Group B, n = 44). Measure of body temperature, bioimpedance analysis, muscular ultrasound, and urinalysis were taken before (t0), immediately after (t_1_), 5’ (t_2_), and 30’ (t_3_) after exercise.

**Results:**

Hydration results in a decreased core temperature; muscular ultrasound showed increased muscle thickness after exercise related to content of body water. Regarding urinalysis, in test H, we found in both groups after exercise a significant decrease of specific urine gravity with significantly lower levels in Group B. We also found a significant increase of pH in the same Group B.

**Conclusions:**

In conclusion all the athletes hydrated with Acqua Lete® showed a positive impact on hydration status after anaerobic exercise with significant decrease of specific urine gravity and a positive effect on pH.

## Background

Scientists and athletes pay particular attention to the strategies of water intake in order to guarantee the best balance of fluids and to improve performance [1,2]. American College of Sports Medicine and the National Athletic Trainers' Association have defined hydration-status founding on urine specific gravity [3,4]. In 1996 the American College of Sport Medicine established the guideline, recently confirmed [5], recommended to preserve an optimal balance of hydration in order to improve performance and to prevent injuries.

Natural, untreated, spring water distinguishes itself from other bottled waters by its specific underground geological origin, its stable composition of minerals and its purity. Mineral waters can have potential beneficial effects on health [6], including bone health and numerous health claims have been made for the benefits arising from the traces of a large number of minerals found in solution [7]. Water alone provides adequate hydration during performance [8]; several researchers have suggested, for instance, that mineral waters, especially those with high concentrations of calcium and bicarbonate, can impact acid–base balance [9] and contribute to the prevention of bone loss [10].

Alkalinizing mineral waters can influence the acid–base equilibrium of the body [11]. Even small changes in pH have crucial effects on cellular function, suggesting that the purposeful consumption of mineral water represents one of the most practical ways to increase the nutritional load of alkali to the body. On the other hand, several studies have shown that alkalinizing mineral waters low in SO_4_^2-^and rich in HCO_3_^-^ had better effects on Ca metabolism and bone resorption markers than waters rich in SO_4_^2-^ and Ca [12].

Acqua Lete® mineral water has calcium concentrations of 314 mg/L, magnesium of 15 mg/L and bicarbonate of 981 mg/L, being a very high calcium and bicarbonate mineral water. The Acqua Lete® exhibits other peculiarities, notably high levels of carbon dioxide, and low contents of sodium and potassium.

Objectives of this study were to examine the relationship between Acqua Lete® intake and total body water, muscle thickness and urinary markers of hydration after short term anaerobic exercise. Based on experimental evidence, we hypothesized that Acqua Lete® mineral water ingestion will correlate with acid–base balance in the body lowering specific urine gravity of athletes and that it can guarantee the effectiveness of a correct hydration during short term exercise.

## Methods

### Protocol

All testing procedures were approved by the institution’s Human Research Ethics committee. Eighty-eight male amateur athletes volunteered to participate in the study. All potential participants attended a familiarization session where details of the test protocol and their time commitment were described. All participants were advised that they were free to withdraw from testing at any time without any adverse consequences. Upon completion of the consent form, participants were randomly divided in two groups (A and B groups) of 44 subjects. Athletes trained (swimming or running) 4–5 hours per week. All the subjects stopped the training and followed a diet without any kind of mineral supplements during the entire period of the study (2 weeks).

Group A : age 34.7 y ± 7.4 (mean ± S.D.); height 178.5 cm ± 5.6; weight 79.6 kg ± 6.9, and Body Mass Index (BMI) 24.6 ± 1.2. Group B : age 33.7 y ± 8.6 (mean ± S.D.); height 174.6 cm ± 5.4; weight 79.6 kg ± 9.6, and Body Mass Index (BMI) 25.7 ± 3.4.

Both groups underwent two experimental trials, performed on an electrically braked ergometer (Bycicle SECA Hamburg, Germany) with a modified repeated Wingate protocol: five bouts of cycling of 60” with a mean speed of 80 RPM and 60” of rest between the sessions. The workload was 85 % of their maximal workload computed in a preliminary session a week before the first Test, with an incremental test on bicycle until exhaustion.

The two Tests were: test C of control, in basal conditions and without hydration the day of trial, for both groups and test H, after one week of controlled hydration with 1.5 L/die of a very low mineral content water in group A and 1.5 L/die of Acqua Lete®, a bicarbonate calcic water with a medium mineral content in group B. Moreover athletes received 750 ml of water using freshly opened bottles one hour before the exercise and 250 ml of water in the following 30 minutes after effort, as recommended by National Athletic Trainer Association [4]. The type of water used was still the very low mineral content water (Group A) and Acqua Lete® (Group B).

Before testing, participants received a physical examination including medical history. In each session of work (Test C and Test H), we measured: body temperature; total body water (TBW), extracellular water (ECW), intracellular water (ICW); muscular size of quadriceps femoris; urinalysis.

The timing of measurements were:

*at rest before the exercise (t*_*0*_*):* body temperature, bioimpedance analysis for TBW, ECW and ICW, muscular ultrasound for detection of muscular size, urinalysis;

*immediately after the last session of exercise (t*_*1*_*):* body temperature;

*5 minute after exercise (t*_*2*_*):* bioimpedance analysis, muscular ultrasound examination;

*30 minutes after exercise (t*_*3*_*):*urinalysis;

### Water analysis

The bicarbonate-rich mineral water Acqua Lete (Acqua Lete®; Società Generale delle Acque Minerali, Pratella, CE, Italy), consumed by the experimental Group B was shipped directly to the testing lab from its bottling facility. The very low mineral content water used for Group A is commonly available throughout Italy; it does not contain significant minerals or electrolytes whatsoever. Very low mineral content and Acqua Lete waters were also analyzed for 15 chemical parameters in our laboratory. Most of the elements were determined by ion chromatography (IC) using a Dionex instrument. A non-acidified aliquot was used to determine pH, electrical conductivity (EC), to titrate alkalinity. The 15 chemical and chemical-physical variables measured on each sample are listed in Table 1. Analytical methods are not further discussed here since they represent standard methods fixed by Italian regulations (IRSA – CNR methods 1994). Results are expressed as mean values ± SD (standard deviation) of three replicate analyses for each water.

**Table 1:** Chemical characteristics of mineral waters used in the study*

**Parameter**	**Measurement unit**	**AcquaLete**®	**Very low mineral content**
Conductivity	mS/cm	1321.40 ± 46.10	17.57 ± 0.91
pH	pH	6.14 ± 0.11	5.00 ± 0.09
Fixed residue	mg/l	878.41 ± 25.21	14.31 ± 0.68
CO_2_	mg/L	1890.12 ± 72.51	15.22 ± 0.77
HCO3^-^	mg/l	981.11 ± 33.82	3.51 ± 0.15
Cl^-^	mg/l	8.24 ± 2.22	0.41 ± 0.02
SO_4_^2-^	mg/l	6.60 ± 0.91	1.40 ± 0.08
NO_3_^-^	mg/l	4.14 ± 0.20	1.91 ± 0.08
Na^+^	mg/l	4.91 ± 0.33	1.21 ± 0.05
K^+^	mg/l	2.10 ± 0.08	0.32 ± 0.01
Ca^++^	mg/l	313.70 ± 9.81	1.11 ± 0.05
Mg^++^	mg/l	15.12 ± 3.92	0.42 ± 0.03
Fe	mg/l	0.02 ± 0.01	< 0.01
Sr^++^	mg/l	0.15 ± 0.01	< 0.1
Li^+^	mg/l	< 0.01	< 0.01

*Each results represents the mean ± SD of three analysis for each water.

### Body temperature

The Measurement of body temperature was made by means of tympanic thermometer Braun ThermoScan.

### Bioimpedance analysis

The qualitative and quantitative appraisal of the body composition was made by means of instrumentation Bodygram AKERN, Florence Italy, which evaluates body and tissue composition, hydration and nutrition status. BIA methods are based on empirical equations based on height, weight and resistance or impedance of the wrist-ankle at 50 kHz, and allows determination of fluid volume and total body water from measurements of resistivity of tissues. We estimated the following parameters: total body water (TBW), extracellular body water (ECW) and intracellular body water (ICW). The examination at T0 was performed fasting from food and drink, whereas at T2 after the controlled hydration.

### Muscle ultrasound

Muscle thickness were determined on the right leg by ultrasonography with a 10 MHz probe with the subject sitting on the examination couch with hips and knees flexed at 90° as reported previously. Muscular ultrasound is a non invasive, available method to detect differences in muscular size after exercise [13]. Subjects were asked to stay relaxed. The same operator performed all measurements at the border between the lower one third and the upper two thirds of the distance between the anterior superior iliac spine and the upper pole of the patella. The measuring point was marked with a marking pen. Measurements were performed just before the exercise test (t_0_), and 5 minute after the end of the cycloergometer test (t_2_). We measured the thickness of the quadriceps femoris (rectus femoris + vastus intermedius) with the probe placed in the transverse plane.

### Urinalysis

The urine was collected in polyethylene containers and mixed with 5 ml/L of a 5 % solution of thymol in isopropanol to preserve the urine. During the collection period, the containers and their contents were maintained at 5 °C. Urine samples were tested for the presence of blood and infection. Nitrite-positive and haematuria samples were discarded. Urine Specific Gravity was evaluated using a refractometer (Atago Digital Urine Specific Gravity Refractometer). Urine pH was recorded using a Rondolino sample changer potentiometer (Mettler Toledo). The color of the urine has been evaluate using a visual staircase. Vogel 1 (yellow urine, yellow pale, yellow clear), Vogel 2 (yellowish urines, reddish, redheads), Vogel 3 (red brownish and brown urines). 2 (yellowish urines, reddish, redheads), Vogel 3 (red brownish and brown urines).

### Statistical analyses

Statistical analysis was performed by SPSS statistical package for Windows, release 17.0 (Chicago, IL, USA). We compared the data collected in each group at every step of work. Statistical significance between group A and group B was evaluated by unpaired samples *T* Test : descriptive statistics were calculated, and values reported as mean ± SD. Statistical significance within group A and group B, comparing Test C and Test H, was also evaluated by Student’s *T* Test for paired samples: descriptive statistics were calculated, and values are reported as mean ± standard deviation. Relationships between the measures collected were calculated with a bivariate correlation measuring the Pearson’s correlation coefficient. Differences were considered statistically significant when P ≤ 0.05.

## Results and discussion

All of the subjects underwent the protocol as described. In Table 1 we reported the features of the mineral waters used in the study.

Tests were performed at an environmental temperature of 19.50 ± 0.53 °C with a wetness of 58.38 ± 0.52 %.

### Test C

In the first test made without hydration, the body temperature showed a significant increase immediately at the end of the cycloergometer test: the athletes started exercise with a mean temperature of 35.9 ± 0.6 °C, reaching at the end of work 36.5 ± 0.4 °C; (p < 0.001). No differences were perceived in total body water distribution, with almost the same levels of ICW and ECW detected before (t_0_) and 5 minute after exercise (t_2_). Conversely significant changes were detected in TBW during the test C (Table 2).

**Table 2:** Total body water (TBW), Extracellular water (ECW) and Intracellular water (ICW) in Test C (control) and in Test H (hydration) before and after exercise*

**Test C**	**TBW**		**ECW**		**ICW**	
	**t**_**0**_	**t**_**3**_	**t**_**0**_	**t**_**3**_	**t**_**0**_	**t**_**3**_
Group A	56.69 ± 1.14^a^	55.30 ± 1.05^a^	40.60 ± 2.48	41.20 ± 2.84	59.40 ± 2.40	58.81 ± 2.84
Group B	57.50 ± 1.80^b^	55.87 ± 0.75^b^	37.76 ± 4.17	37.46 ± 2.82	62.24 ± 4.17	62.54 ± 2.82
**Test H**	**TBW**		**ECW**		**ICW**	
	**t**_**0**_	**t**_**3**_	**t**_**0**_	**t**_**3**_	**t**_**0**_	**t**_**3**_
Group A	57.83 ± 3.75	57.43 ± 5.01	40.85 ± 2.87	40.57 ± 2.42	59.15 ± 2.87	59.43 ± 2.42
Group B	57.84 ± 2.26	57.37 ± 3.11	38.47 ± 1.11^c^	37.10 ± 1.04^c^	61.53 ± 1.14^d^	62.94 ± 0.94^d^

^*^values are expressed in percentage (%).

Data are expressed as mean ± SD: n = 44.

Mean values were significantly different from resting values (t_0_): ^a and b^p < 0.001; ^c and d^p < 0.05.

Ultrasonography performed at rest (t_0_) and 5’ after the Wingate test (t_2_) showed in both groups a variation of muscular thickness, consistent with our previous study [11]. (Group A: 29.94 ± 3.89 mm *vs* 32.29 ± 3.13 mm: p = 0.00); Group B: 30.56 ± 3.30 mm *vs* 33.08 ± 2.89 mm: p = 0.00).

**Figure 1:**
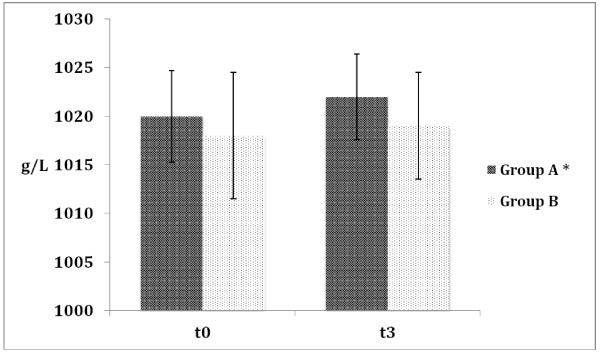
Urinary specific gravity detected in Test C (Control) before and after exercise*. *Data are expressed as mean ± SD; n = 44; Group A: 1020 ± 4.7 (t_0_) vs 1022 ± 4.4 (t_3_): p = < 0.05 Group B: 1018 ± 6.5 (t_0_) vs 1019 ± 5.5(t_3_), p = ns.

Urinalysis collected at t_0_ and t_3_ showed no significant difference in colour; we observed a decrease of urinary pH at t_2_ (Table 3), as expected after anaerobic exercise, whereas specific urinary gravity after effort (Figure 1) showed a significant increase (Group A: 1020 ± 4.7 g/L *vs* 1022 ± 4.4 g/L; p = <0.001; Group B: 1018 ± 6.5 g/L *vs* 1019 ± 5.5 g/L; p = ns). Data on urine pH and specific gravity between the two groups were compared. The values were not different between the two groups.

**Table 3:** Urine pH detected in Test C (control) and in Test H (hydration) before and after Exercise*

**Test C**	**t**_**0**_	**t**_**2**_
Group A	5.6 ± 0.2^a^	5.3 ± 0.1^a^
Group B	5.6 ± 0.4	5.4 ± 0.5
**Test H**	**t**_**0**_	**t**_**2**_
Group A	5.5 ± 0.8	5.4 ± 0.9
Group B	5.4 ± 0.2^b^	5.7 ± 0.1^b^

* Data are expressed as mean ± SD, n = 44.

Mean values were significantly different: ^a and b^p < 0.05.

### Test H

The body temperature showed an increase t_0_-t_1_ in test C (35.9 ± 0.4 °C *vs* 36.4 ± 0.4 °C; p = <0.001). Bioimpedance analysis performed after hydration (Table 2), showed no difference in group A, whereas in group B we found a slight but significant decrease of ECW at rest and a concomitant increase of ICW. After exercise group B showed a shift of body water, from extracellular to intracellular compartment.

Ultrasonography detected an increase in muscular thickness, in test H. (Group A: 29.93 ± 3.89 mm *vs* 32.00 ± 3.61 mm; Group B: 30.84 ± 3.47 mm *vs* 32.82 ± 2.72 mm).

**Figure 2:**
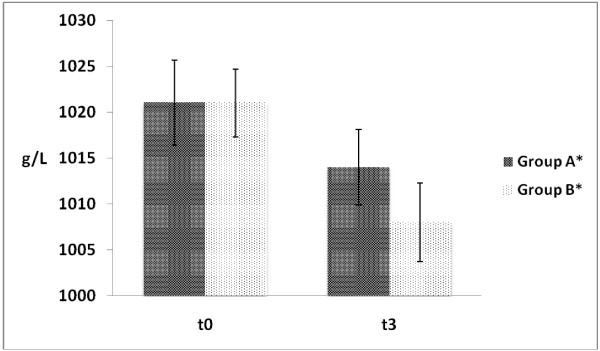
**Urinary specific gravity detected in Test H (test with hydration) before (t**_**0**_**) and 30’ after exercise (t**_**3**_)*. *Data are expressed as mean ± SD; n = 44; Group A: 1021 ± 4.6 (t_0_) vs 1014 ± 4.1(t_3_), p = < 0.05 Group B: 1021 ± 3.7 (t_0_) vs 1008 ± 4.3 (t_3_), p = < 0.05 Group A (t_3_) vs Group B (t_3_) = p < 0.05.

In athletes hydrated with Acqua Lete urine pH was more alkaline than in those who drank very low mineral content water (Table 3). The specific gravity of the urine after effort sustained a significant and similar decrease in the two groups but subjects who drank Acqua Lete mineral water (Group B) showed a significantly lower mean values of specific urinary gravity when compared with athletes belonging to Group A (Group A 1014 ± 4.1 g/L vs Group B 1008 ± 4.3 g/L - Figure 2).

Many studies used Wingate Test and modified Wingate Test [14], to assess physiological responses to anaerobic exercise. In our study we evaluated the response to anaerobic exercise before and after hydration with a bicarbonate-calcic mineral water, named Acqua Lete, compared to a very low mineral content water (dry residues 14.3 mg/L).

A modest increase in core body temperature occurred despite subjects performed at a moderately high exercise intensity for a short time, although there are not univocal conclusions in the literature about the relation between core temperature, intensity of exercise and hydration status [15]. However some studies reported increase of core temperature after Wingate test, with a fatigue index higher when core temperature values are highest [16]. The exact mechanism of fatigue is not known; but presumably it is a complex interplay between both peripheral and central factors: the mechanism is probably mediated by catecholamines dopamine and noradrenaline. [17].

Other studies reported increase of temperature after light exercise, as the warm-up, depending on the duration of exercise [18]. The relationship between level of hydration and core temperature has been widely studied and, although it is well documented that dehydration increases body temperature during exercise [19], many studies agree that hyperhydration provides no thermoregulatory advantage over the maintenance of euhydration during exercise [20]. In our study we found a slight but significant difference in body temperature after exercise between Test C and Test H (36.5 ± 0.4 °C *vs* 36.4 ± 0.4 °C; p = <0.001), with lower values after hydration, confirming that the euhydration obtained in the second test ensured a better thermoregulatory homeostasis.

Body composition assessment is useful in a variety of clinical settings to gain information about nutritional condition and the status of body fluid compartments. Bioimpedance analysis (BIA) is an attractive technique for the purpose, because it is safe, non-invasive, inexpensive and easy to use. Previous studies have characterized the accuracy of bioimpedance analysis [21] and have reported difference in total body water before and after effort, due to a shift from extracellular to intracellular compartment consequent to modification of cellular osmolarity after energy depletion [22,23]. During exercise, the elevated metabolic activity within the cell, leads to increased osmotic pressure, stimulates an influx of fluid into the intracellular compartment to re-establish an osmotic equilibrium [24].

Although changes in TBW are reported in the literature as a consequence of long-term exercise [25], we found significant change of TBW in both groups, when not hydrated. Conversely, after hydration both groups showed a similar total body water, but different distribution of ECW and ICW: Group B, hydrated with a bicarbonate calcic mineral water (Acqua Lete®), showed a significant shift of water through intracellular compartiment. This group reached at peak of exercise a higher level of blood lactate (9.8 ± 0.6 mmol/L *vs* 7.4 ± 0.8 mmol/L; p < 0.05), leading to a change of intracellular pH and mediating cellular osmolality, which may be responsible for the increased volume of water in the intracellular space [26].

An ultrasound examination of both groups showed a similar increase of muscle thickness *5* minute after the end of the cycloergometer test, with a mean increase of 2.14 ± 1.06 mm in Group A and 2.55 ± 1.22 in Group B. Changes in size and muscle architecture, reported in a number of studies, were related to the biochemical changes which occurred with muscle fatigue [27]. In a previous study we found a significant increase of muscle thickness after cycloergometer test, bound to a variation of muscle architecture [13] probably as a consequence of muscle oedema. However the increased muscle thickness may be also resulting from a slowing of muscle relaxation due to intracellular accumulation of Ca^++^ and H^+^: in fact the elevation of the Ca^++^-dependent proteolytic pathway degrades structural and contractile proteins, and depression in pH reduces the rate of cross bridge detachment [28].

After hydration we also found in both groups an interesting correlation between the increase of ICW and the thickness of quadriceps (Group A: r = 0.957, p < 0.001; Group B: r = 0.454, p < 0.05): in this case the increased volume of quadriceps seems to be due to a higher content of cellular water. (Group A = mean increase of 2.35 ± 1.27 vs Group B 2.52 ± 0.91). We did not find this relation in Test C: one possible explanation is that in the control test the increase of thickness was mainly due to the lack of relaxation, possibly the consequence of mild dehydration on neuro-muscular control [29].

Urinalysis assesses hydration status, particularly with urine osmolarity, specific gravity and colour [30]. In our study we evaluated specific urine gravity, pH and colour before (t_0_) and 30’ after the end of the cycloergometer test (t_3_) in both sessions (without and with hydration).

When the groups were tested without hydration, we found in both groups a slight but significant increase of urine gravity after exercise. The date had the same course in both groups thus reaching a significant difference in group A. Even if a more complete study which take account all the aspects of fluid balance (urine volume osmolarity and hematocrit) could give more detail, We think that this result might be due to different hydration status (TBW) in the groups as described in Table 2. Conversely, in test H the controlled hydration imposed during the week before the test, lead to an equal TBW at rest. Anyway we supposed decreasing of urinary specific gravity after acute hydration, but we found that group B reached after exercise a significantly lower level than group A (1008.1 ± 4.3 g/L *vs* 1014.6 ± 4.1 g/L; p = <0.001). Both groups were well hydrated, but group B reading less than 1.010 reflected a better hydrated condition than the group A [5].

This result can be attributed to the specific chemical composition of waters used in Test H: the very low mineral content water had low levels of calcium and bicarbonate and a fixed residue of 14.3 mg/L; the Acqua Lete® water (fixed residue 878.8 mg/L) with modest contents of sodium (4.9 mg/L), potassium (2.1 mg/L) and sulphate (6.6 mg/L) had significant contents of bicarbonate (range values of 981.1), calcium (313.7 mg/L) and magnesium (15.1 mg/L), belongs to the group of the bicarbonate-calcics.

The specific gravity is dependent on the number and weight of solute particles constituted mainly of urea and electrolytes. In physiological conditions the greater absorption of water induce a lower concentration of solutes, producing urine with a low specific gravity, which indicates better capacity to retain water as we found in Group B. Moreover, consumption of mineral waters rich in magnesium and bicarbonate can increase urinary pH, magnesium, and citrate and decrease calcium oxalate concentration [31].

In the present study, when compared with the consumption of the very low mineral content bottled water, hydration with Acqua Lete® mineral water was associated with a significant increase in urine pH. Previous research by König et al. [32] demonstrated that consumption of a mineral-rich supplement significantly increased urinary pH. Similarly, Heil [9] (2010) showed that mineral-rich bottled water with alkalinizant supplement improved acid–base balance and hydration status. The observations from these studies are consistent with the changes in urine observed in the present study for Group B. Moreover in a previous study [26] we found that the better hydration status improved the recovery after exercise in both groups of athletes, with a rate of decrease of lactate higher in test H respect the test C. Besides the specificity of the Acqua Lete water, have affected the increase of lactate at peak of exercise and the restore after exercise, leading to minimal, but significantly lower levels of [La^-^ after effort.

## Conclusions

To date most of the studies focused on the maintenance of better hydration status during strenuous exercise, whereas little has been written on useful strategies of rehydration in short term exercise, when water loss is minimal and other aspects of recovery may be taken into account. The results of our study confirm that in short term exercise, a correct hydration is important as well as in long term exercise and confirm our hypothesis that Acqua Lete® mineral water intake is correlated with the increase of urinary pH and with a lower urine specific gravity in amateur athletes, therefore it may be a valuable nutritional vector for influencing hydration status in athletes.

## Abbreviations

Test C, Control test; Test H, Test with hydration; BMI, Body Mass Index; TBW, Total Body Water; ECW, Extracellular Water; ICW, Intracellular Water; IC, Ion Chromatography; EC, Electrical Conductivity; BIA, Bioimpedance Analysis.

## Competing interest

We declare that no conflict of interest. We have no financial or other interest in the product or distributor of the product.

## Author’s contribution

Paola Brancaccio, participated the design of the study, performed the statistical analysis, the interpretation of data and drafted the manuscript, Francesco Mario Limongelli, have given final approval of the version, Iride Paolillo, participated to the acquisition of data and carried out urinalysis, bioimpedance analysis and muscle ultrasound, Antonio D’Aponte, participated to the acquisition of data and carried out the Wingate test, Vincenzo Donnarumma, carried out all the laboratory analysis, Luca Rastrelli, performed the water analysis, participated the interpretation of data, drafted the manuscript and given final approval of the version. All authors read and approved the final manuscript.

## Limitation of the study

We did not afford a complete assessment of hydration status, because the short duration of exercise and the lack of sweating did not allow to appreciate changes in body weight. A more complete study which take account all the aspects of fluid balance (urine volume osmolarity and hematocrit) and a complete diet, could give more detail and better indication on type of water to use in different type of exercise.
